# Botulinum Toxin Physiology in Focal Hand and Cranial Dystonia

**DOI:** 10.3390/toxins4111404

**Published:** 2012-11-20

**Authors:** Barbara Illowsky Karp

**Affiliations:** National Institute of Neurological Disorders and Stroke, National Institutes of Health, 9000 Rockville Pike, Bethesda, MD 20892, USA; Email: karpb@ninds.nih.gov; Tel.: +1-301-496-0150; Fax: +1-301-480-2983

**Keywords:** dystonia, blepharospasm, Meige syndrome, writer’s cramp, botulinum toxin, physiology

## Abstract

The safety and efficacy of botulinum toxin for the treatment of focal hand and cranial dystonias are well-established. Studies of these adult-onset focal dystonias reveal both shared features, such as the dystonic phenotype of muscle hyperactivity and overflow muscle contraction and divergent features, such as task specificity in focal hand dystonia which is not a common feature of cranial dystonia. The physiologic effects of botulinum toxin in these 2 disorders also show both similarities and differences. This paper compares and contrasts the physiology of focal hand and cranial dystonias and of botulinum toxin in the management of these disorders.

## 1. Introduction

Focal hand dystonia and cranial dystonia share the phenotypic features of uncontrolled involuntary muscle contraction leading to abnormal postures characteristic of all dystonias. They differ, however, not only in the anatomic distribution of focal dystonia symptoms, but also in age of onset, prevalence, gender predilection, and pathways implicated in their pathophysiology. Heterogeneity in the focal dystonias have led some to question whether they are etiologically related [1]. While the safety and efficacy of botulinum toxin for the treatment of these focal dystonias have been established through controlled clinical trials and over 20 years of use [2], their response to botulinum toxin treatment similarly reveals both shared features and divergent features. Comparing and contrasting these distinct yet overlapping disorders may provide clues to the nature of the dystonia and the action of botulinum toxin in their treatment. 

## 2. Physiology of Focal Hand Dystonia and Cranial Dystonias

Focal hand dystonia is characterized by abnormal muscle contraction that leads to involuntary movements and postures and subsequently to hand dysfunction. When dystonia affects the hand, it can cause remarkable disability even if the symptoms of muscle tightness and observable postural changes appear mild. A notable feature of focal hand dystonia is “task specificity,” in which the dystonia is only elicited by particular tasks while other actions, including those using the same muscles, are spared. The terms “writer’s cramp” and “musician’s cramp” reflect this task specificity. Another distinguishing feature of focal hand dystonia is its association with hand overuse. From its earliest descriptions in the 18th century, focal hand dystonia was reported as most likely to arise in those whose occupations or hobbies include repetitive fine motor tasks [3]. Limb overuse is clearly not the sole risk factor for focal hand dystonia as many who use their hands extensively such as professional musicians, do not develop dystonia, while it is often present in those with only average hand use. An underlying genetic or other predisposition may be a necessary substrate [1]. 

Focal hand dystonia usually first presents between the age of 30 and 50. Unlike other focal dystonias which are more common in women, writer’s cramps affects men and women approximately equally and musician’s cramp shows a male predominance [4]. 

Key studies of the physiology of dystonia have focused on focal hand dystonia as it offers several advantages over other forms of dystonia. First, hand cortical sensory and motor representations can be readily identified on brain imaging and selectively targeted for such techniques as transcranial magnetic stimulation (TMS). The task specificity of focal hand dystonias permits separation of dystonic movements, hand movements that do not elicit dystonia and resting conditions. Finally, the frequently unilateral nature of focal hand dystonia provides an unaffected hand for comparison. 

Multiple research methodologies have demonstrated a loss of various types of inhibition in focal hand dystonia, including impaired reciprocal, intracortical, surround and interhemispheric inhibition [5]. For example, stimulation of Ia afferent nerves of an agonist muscles suppresses EMG activity in the antagonist muscles, known as reciprocal inhibition [6]. Reciprocal inhibition has been shown to be deficient in both the dystonic and non-dystonic hand which may be attributed to loss of descending inhibitory influences from higher cerebral structures [7]. Aberrant muscle spindle activity may also contribute to the muscle overflow and co-contraction characteristic of dystonia [8]. Intracortical inhibition can be studied with paired pulse transcranial magnetic stimulation (TMS), in which a first subthreshold TMS stimulus suppresses the amplitude of the muscle electromyographic (EMG) response to a second suprathreshold TMS pulse. Such TMS techniques have shown impairment of intracortical inhibition, sometimes bilaterally and sometimes unilaterally and specific to the dystonic hand [5]. TMS also reveals diminished transcallosal inhibition [9] and diminished surround inhibition in which activation of muscles need to perform a task fails to inhibit activity in surrounding muscles not involved in the task being performed [10]. Vibration activates muscle spindle 1A afferents, induces muscle contraction (termed the “tonic vibration reflex”) more frequently in those with FHD than in healthy controls and can provoke a patient’s dystonic symptoms [11]. The effect of vibration can be blocked by lidocaine, consistent with a role for muscle spindles in FHD. Additional findings that may stem at least in part from deficient inhibition include abnormal cortical excitability and excessive sensorimotor plasticity [5,12]. Loss of inhibition may also account for increased cerebral activity in sensorimotor cortex, premotor areas and cerebellum during tasks that elicit the dystonia, while decreased activity is found in primary sensorimotor cortex and supplementary motor areas when the dystonia is not active on fMRI and PET scans [13,14,15,16,17]. Resting state functional MRI shows abnormal connectivity in sensorimotor and default mode networks [18]. Studies in focal hand dystonia have also led to the recognition of sensory dysfunction in dystonia. Patients with focal hand dystonia have distorted sensory maps and defects in temporal and spatial discrimination [19,20] as well as impaired sensorimotor integration [15,18]. Subcortical white matter changes can be identified as well in focal hand dystonia. Diffusion tensor magnetic resonance imaging identified changes in the fiber tracts in the posterior limb of the internal capsule, connecting sensorimotor cortex to subcortical structures, including thalamocortical and corticostriatal fibers [21].

Cranial dystonia is characterized by abnormal movements of various combinations of the muscles of facial expression, muscles of mastication, lingual muscles and, at times, laryngeal and pharyngeal muscles. The most common cranial dystonia is blepharospasm, characterized by uncontrolled contraction of orbicularis oculi muscles bilaterally resulting in forced eye closure which, when severe, leads to functional blindness. Dystonia may be isolated to the lower cranial muscles (oromandibular dystonia) or lower cranial musculature dystonia may combine blepharospasm (Meige syndrome) [22,23,24,25]. Dystonic spasm of the lower cranial muscles causes involuntary jaw closing, jaw opening dystonia, or a combination of both movements. There may also be lateral jaw deviation, grimacing, and tongue writhing. Speech, chewing and swallowing are often impaired and cranial dystonia is often socially disfiguring. Cranial dystonias usually arise in midlife and affect women more than men. Blepharospasm and oromandibular dystonias largely arise without a background of specific muscle overuse. Reported precipitants include eye disease, such as dry eyes, blepharitis or keratoconjunctivitis in those with blepharospasm [26,27] and jaw trauma or dental procedures in those with oromandibular dystonia [28]. Although less frequently than in focal hand dystonia, the abnormal movements of oromandibular dystonia may be task-specific. For example, dystonic movements may only be present on playing a wind or brass instrument in musicians with embouchure dystonia [29]. 

Cranial dystonia shares with focal hand dystonia evidence of prolonged muscle contraction, antagonist cocontraction [30] and loss of central inhibition. The loss of central inhibition can be demonstrated by the blink reflex studies. In the blink reflex, a first subthreshold electrical stimulus to the supraorbital nerve inhibits the orbicularis oculi response to a second suprathreshold stimulus. The degree of inhibition varies with the time interval between the 2 impulses. Patients with blepharospasm have increased excitability, indicating a loss of inhibition [31]. Similar loss of inhibition can be demonstrated with the masseter inhibitory reflex in cranial dystonia [32]. The cortical silent period, a lapse in EMG activity due to TMS, is abnormally short in both dystonic and non-dystonic muscles in oromandibular dystonia [33], consistent with increased cortical excitability in those patients. 

Similar to focal hand dystonia where increased activity is found in sensorimotor and premotor territories when the dystonia is active [13,14], those with orofacial dystonia may have relative overactivity in somatosensory cortex [34]. Patients with blepharospasm have greater activation in visual cortex, anterior cingulate cortex, motor cortex, thalamus and cerebellum than those without blepharospasm during both spontaneous and voluntary blinking [35]. In musicians with embouchure dystonia, sensorimotor overactivity was seen both with a task that activated the dystonia and a similar task that did not activate the dystonia [29].

Sensory pathways are implicated in cranial dystonia as well as limb dystonia. Those with cranial dystonia often have pain or discomfort and may respond to a sensory trick (“geste antagoniste”), in which a simple touch or gesture temporarily relieves the dystonia. The efficacy of the geste may be mediated by transient trigeminal sensory gating [32]. Abnormal sensory representation, temporal discrimination, sensory processing and sensorimotor integration have been demonstrated for cranial dystonia [36,37,38]. Patients with blepharospasm have decreased activation of primary and secondary somatosensory territories in response to touch stimuli to the face [38]. Regional cerebral blood flow in response to vibration stimuli placed on the face was decreased bilaterally in primary sensory areas in patients with blepharospasm. The decrease in brain activity was best seen with stimulation of areas affected by the dystonia; the decrease was less dramatic in response to hand vibration [39]. Diffusion tensor imaging failed to show white matter differences in patients with blepharospasm compared to healthy controls [40].

## 3. Physiology of Botulinum Toxin in the Treatment of Focal Hand Dystonia and Cranial Dystonias

Studies of the physiologic effects of botulinum toxin in treating dystonia have focused on patients receiving serotype A toxin. It is not known if the other clinically available botulinum toxin serotype, type B, has identical effects.

Botulinum toxin is the current mainstay of treatment for both focal hand dystonia and cranial dystonias [2]. Rehabilitation approaches, such as physical therapy, occupational therapy or speech therapy rarely bring substantial or sustained benefit. Oral medications commonly used for dystonia including anticholinergics, dopamine depletors, muscle relaxants and benzodiazepines are likewise usually at most mildly successful and are associated with significant side effects. Deep brain stimulation is being explored for severe cases of focal hand dystonia and cranial dystonias, but its use has not yet been established. 

Blepharospasm was the first dystonia to be treated with botulinum toxin [41,42]. For blepharospasm, injections are directed to the eye closing muscle-orbicularis oculi. Proper selection of muscles is not as straightforward for other dystonias. The most critical and most challenging aspect of using botulinum toxin for the treatment of focal hand and oromandibular dystonias is determining which muscles are primarily involved in the dystonia and which may be compensating for the dystonia and should not be injected. Muscles are generally chosen for injection based on clinical evaluation and patient report. When selecting muscles for injection, it is important to observe the patient during actions that elicit the dystonia, such as writing or playing their musical instrument [43]. EMG may be helpful in identifying overcontraction of muscles that are deep or not apparent on inspection. The dose of toxin needed for these conditions reflects the size and strength of the muscles involved. Hand intrinsic and facial muscles typically require only small doses. Strong muscles, such as jaw muscles, require a higher dose of toxin than might be expected based solely on muscle mass. At least 90% of patients with blepharospasm benefit from botulinum toxin [44,45]. Oromandibular dystonias often do not respond as well and it has been noted that botulinum toxin may be more effective for jaw closing dystonia than jaw opening dystonia [46,47]. Although about 80% of those with focal hand dystonia have at least mild benefit from botulinum toxin initially, only 20%–30% consider the benefit sufficient to continue injections more than a few years [43]. Benefit in both cranial dystonias and focal hand dystonias can be sustained for years with repeated injection [47,48].

To treat dystonia, botulinum toxin is injected intramuscularly. Botulinum toxin acts at the molecular level at the neuromuscular junction, where it binds to and is internalized by the presynaptic neuron. In the cell, botulinum toxin cleaves SNARE complex proteins and prevents acetylcholine exocytosis, resulting in denervation and weakness in exposed muscles [49]. The intent when botulinum toxin was first developed as treatment was to titrate the toxin effect—to weaken dystonic muscles sufficiently to eliminate dystonic over-contraction without inducing paralysis. Muscle weakening cannot, however, be the sole explanation for the efficacy of botulinum toxin in treating dystonia. Many patients have improvement in dystonia with minimal, if any, detectable weakness while others have substantial weakness without improvement in dystonia. 

The efficacy of botulinum toxin in both limb and cranial dystonia may largely be due to its peripheral neuromuscular junction blockade and weakening of over-contracting muscles, however, there is also evidence of central effects. The mechanism by which botulinum toxin exerts influence on central motor control systems is uncertain. Animal models have shown that botulinum toxin undergoes retrograde and transynaptic transport so that direct effects on the spinal cord and brain are possible [50,51]. Direct effects may be unlikely, however, as the toxin reaches the nerve soma in only small amounts and the toxin may be inactivated during retrograde transport [50,52]. Botulinum toxin blocks cholinergic nerve endings in muscle spindles as well as at the neuromuscular junction, demonstrated by the fact that botulinum toxin can lessen the tonic vibration response (TVR) in patients with limb dystonia. Seven months after toxin injection, the TVR remains depressed, even when maximal muscle strength has been regained, demonstrating a sustained intrafusal effect even when extrafusal effects have worn off [53]. Modulation of spindle afferent input to the central nervous system likely contributes to botulinum toxin’s efficacy in dystonia and it has been suggested the central effects of the toxin are largely explainable by the toxin’s modulation of peripheral afferent input to the central nervous system [8,54]. 

In limb dystonia, botulinum toxin injection can temporarily reverse some central physiologic abnormalities including those in reciprocal inhibition, cortical motor map distortion and intracortical inhibition [55,56,57,58]. The duration of effect on these physiological parameters often parallels the duration of clinical efficacy. Botulinum toxin does not appear to modify or reverse abnormal cortical activation in patients with focal hand dystonia evaluated by TMS or PET scan [59,60,61], but may be able to alter aberrant plasticity. Motor cortex plasticity can be assessed by paired associative stimuli (PAS) in which peripheral nerve and transcranial magnetic stimulation of the contralateral motor cortex combine to alter the excitability of the motor cortex. Using TMS motor evoked potentials of the hand as a measure of motor cortical excitability, patients with cervical dystonia in combination with hand dystonia or dystonic tremor were found to have increased PAS-induced excitability [62]. One month after botulinum toxin injections to the neck muscles only, PAS no longer facilitated the motor evoked potential response in hand muscles. Abnormal excitability returned as the clinical effects of the toxin wore off 3 months later. Botulinum toxin was found to reverse white matter abnormalities detected by diffusion tensor imaging in cervical dystonia [63], but the issue has not yet been studied in focal hand dystonia. 

Central effects of botulinum toxin have been more difficult to demonstrate in cranial dystonia. Several researchers have evaluated the effect of botulinum toxin on the decreased brainstem inhibition, as indicated by abnormal blink reflex recovery curves, in patients with blepharospasm and oromandibular dystonia. These studies show that botulinum toxin does not reverse the enhanced excitability of the blink reflex R2 recovery curve, suggesting that it has no effect on brainstem interneuron excitability even when the toxin is effectively treating the dystonic symptoms [33,64,65], although it has also been suggested that current techniques may not be adequately sensitive to detect such changes [54]. 

Botulinum toxin has similarly failed to show effects on abnormal cortical excitability and plasticity in patients with cranial dystonia. While a single study found that botulinum toxin reduced enhanced plasticity [66], another found that it did not [67]. The lack of effect on cortical excitability in cranial dystonia is supported by the failure of botulinum toxin to reverse the abnormally shortened cortical silent period in blepharospasm [33] and by its lack of effect on decreased sensorimotor cortex activity assessed by fMRI [38].

Botulinum toxin effects on spinal cord excitability in limb dystonia, as demonstrated by its effects on reciprocal inhibition, cannot play a role in response of facial muscles to injection since the spinal cord is not involved in facial muscle innervation. The role of spindle afferents in cranial dystonia and its response to botulinum toxin is uncertain: while early anatomy studies indicated that cranial muscles lack muscle spindles [33], more recent studies have reported that spindles can be found in almost all cranial muscles [8].

## 4. Conclusions

Focal hand dystonia and cranial dystonia share physiological features. Phenotypically, both show abnormal muscle activity with prolonged contraction and cocontraction of antagonist muscle. Loss of inhibition, found in both, is expressed as impaired reciprocal, intracortical, interhemispheric and surround inhibition in focal hand dystonia and as impaired brainstem and intracortical inhibition in cranial dystonia. Abnormalities of cortical activation, sensory processing and sensorimotor integration have also been implicated in both. 

Botulinum toxin is effective in both focal hand dystonia and cranial dystonias. In focal hand dystonia, the response to botulinum toxin may be at least in part mediated through its effects on muscle spindle afferents, which in turn modulate central function. The role of peripheral afferent input in cranial dystonia and response to botulinum toxin is less uncertain. Further research is needed to understand the mechanism of efficacy of botulinum toxin in these disorders. 

## References

[1] Defazio G., Berardelli A., Hallett M. (2007). Do primary adult-onset focal dystonias share aetiological factors?. Brain.

[2] Simpson D.M., Blitzer A., Brashear A., Comella C., Dubinsky R., Hallett M., Jankovic J., Karp B., Ludlow C.L., Miyasaki J.M. (2008). Assessment: Botulinum neurotoxin for the treatment of movement disorders (an evidence-based review): Report of the therapeutics and technology assessment subcommittee of the American Academy of Neurology. Neurology.

[3] Gowers W.R. (1888). A Manual of Diseases of the Nervous System.

[4] Brandfonbrener A.G., Robson C. (2004). Review of 113 musicians with focal dystonia seen between 1985 and 2002 at a clinic for performing artists. Adv. Neurol..

[5] Hallett M. (2011). Neurophysiology of dystonia: The role of inhibition. Neurobiol. Dis..

[6] Day B.L., Marsden C.D., Obeso J.A., Rothwell J.C. (1984). Reciprocal inhibition between the muscles of the human forearm. J. Physiol..

[7] Chen R.S., Tsai C.H., Lu C.S. (1995). Reciprocal inhibition in writer’s cramp. Mov. Disord..

[8] Rosales R.L., Dressler D. (2010). On muscle spindles, dystonia and botulinum toxin. Eur. J. Neurol..

[9] Koch G., Schneider S., Baumer T., Franca M., Munchau A., Cheeran B., del Olmo F.M., Cordivari C., Rounis E., Caltagirone C. (2008). Altered dorsal premotor-motor interhemispheric pathway activity in focal arm dystonia. Mov. Disord..

[10] Sohn Y.H., Hallett M. (2004). Disturbed surround inhibition in focal hand dystonia. Ann. Neurol..

[11] Kaji R., Rothwell J.C., Katayama M., Ikeda T., Kubori T., Kohara N., Mezaki T., Shibasaki H., Kimura J. (1995). Tonic vibration reflex and muscle afferent block in writer’s cramp. Ann. Neurol..

[12] Weise D., Schramm A., Stefan K., Wolters A., Reiners K., Naumann M., Classen J. (2006). The two sides of associative plasticity in writer’s cramp. Brain.

[13] Odergren T., Stone-Elander S., Ingvar M. (1998). Cerebral and cerebellar activation in correlation to the action-induced dystonia in writer’s cramp. Mov. Disord..

[14] Pujol J., Roset-Llobet J., Rosines-Cubells D., Deus J., Narberhaus B., Valls-Sole J., Capdevila A., Pascual-Leone A. (2000). Brain cortical activation during guitar-induced hand dystonia studied by functional MRI. Neuroimage.

[15] Wu C.C., Fairhall S.L., McNair N.A., Hamm J.P., Kirk I.J., Cunnington R., Anderson T., Lim V.K. (2010). Impaired sensorimotor integration in focal hand dystonia patients in the absence of symptoms. J. Neurol. Neurosurg. Psychiatry.

[16] Havrankova P., Walker N.D., Operto G., Sieger T., Vymazal J., Jech R. (2012). Cortical pattern of complex but not simple movements is affected in writer’s cramp: A parametric event-related fMRI study. Clin. Neurophysiol..

[17] Ibanez V., Sadato N., Karp B., Deiber M.P., Hallett M. (1999). Deficient activation of the motor cortical network in patients with writer’s cramp. Neurology.

[18] Mohammadi B., Kollewe K., Samii A., Beckmann C.F., Dengler R., Munte T.F. (2012). Changes in resting-state brain networks in writer’s cramp. Hum. Brain Mapp..

[19] Bara-Jimenez W., Shelton P., Hallett M. (2000). Spatial discrimination is abnormal in focal hand dystonia. Neurology.

[20] Bara-Jimenez W., Shelton P., Sanger T.D., Hallett M. (2000). Sensory discrimination capabilities in patients with focal hand dystonia. Ann. Neurol..

[21] Delmaire C., Vidailhet M., Wassermann D., Descoteaux M., Valabregue R., Bourdain F., Lenglet C., Sangla S., Terrier A., Deriche R. (2009). Diffusion abnormalities in the primary sensorimotor pathways in writer’s cramp. Arch. Neurol..

[22] Tolosa E., Kulisevsky J., Fahn S. (1988). Meige syndrome: Primary and secondary forms. Adv. Neurol..

[23] Tan E.K., Jankovic J. (1999). Botulinum toxin A in patients with oromandibular dystonia: Long-term follow-up. Neurology.

[24] Marsden C.D. (1976). Blepharospasm-oromandibular dystonia syndrome (Brueghel’s syndrome). A variant of adult-onset torsion dystonia?. J. Neurol. Neurosurg. Psychiatry.

[25] Bakke M., Larsen B.M., Dalager T., Moller E. (2012). Oromandibular dystonia-functional and clinical characteristics: A report on 21 cases. Oral Surg. Oral Med. Oral Pathol. Oral Radiol..

[26] Martino D., Defazio G., Alessio G., Abbruzzese G., Girlanda P., Tinazzi M., Fabbrini G., Marinelli L., Majorana G., Buccafusca M. (2005). Relationship between eye symptoms and blepharospasm: A multicenter case-control study. Mov. Disord..

[27] Defazio G., Abbruzzese G., Aniello M.S., Bloise M., Crisci C., Eleopra R., Fabbrini G., Girlanda P., Liguori R., Macerollo A. (2011). Environmental risk factors and clinical phenotype in familial and sporadic primary blepharospasm. Neurology.

[28] Sankhla C., Lai E.C., Jankovic J. (1998). Peripherally induced oromandibular dystonia. J. Neurol. Neurosurg. Psychiatry.

[29] Haslinger B., Altenmuller E., Castrop F., Zimmer C., Dresel C. (2010). Sensorimotor overactivity as a pathophysiologic trait of embouchure dystonia. Neurology.

[30] Mascia M.M., Valls-Sole J., Marti M.J., Sanz S. (2005). Chewing pattern in patients with Meige’s syndrome. Mov. Disord..

[31] Berardelli A., Rothwell J.C., Day B.L., Marsden C.D. (1985). Pathophysiology of blepharospasm and oromandibular dystonia. Brain.

[32] Berardelli A., Curra A. (2002). Pathophysiology and treatment of cranial dystonia. Mov. Disord..

[33] Allam N., Fonte-Boa P.M., Tomaz C.A., Brasil-Neto J.P. (2005). Lack of effect of botulinum toxin on cortical excitability in patients with cranial dystonia. Clin. Neuropharmacol..

[34] Dresel C., Haslinger B., Castrop F., Wohlschlaeger A.M., Ceballos-Baumann A.O. (2006). Silent event-related fMRI reveals deficient motor and enhanced somatosensory activation in orofacial dystonia. Brain.

[35] Baker R.S., Andersen A.H., Morecraft R.J., Smith C.D. (2003). A functional magnetic resonance imaging study in patients with benign essential blepharospasm. J. Neuroophthalmol..

[36] Bradley D., Whelan R., Kimmich O., O’Riordan S., Mulrooney N., Brady P., Walsh R., Reilly R.B., Hutchinson S., Molloy F. (2012). Temporal discrimination thresholds in adult-onset primary torsion dystonia: An analysis by task type and by dystonia phenotype. J. Neurol..

[37] Hirata Y., Schulz M., Altenmuller E., Elbert T., Pantev C. (2004). Sensory mapping of lip representation in brass musicians with embouchure dystonia. Neuroreport.

[38] Dresel C., Bayer F., Castrop F., Rimpau C., Zimmer C., Haslinger B. (2011). Botulinum toxin modulates basal ganglia but not deficient somatosensory activation in orofacial dystonia. Mov. Disord..

[39] Feiwell R.J., Black K.J., McGee-Minnich L.A., Snyder A.Z., MacLeod A.M., Perlmutter J.S. (1999). Diminished regional cerebral blood flow response to vibration in patients with blepharospasm. Neurology.

[40] Fabbrini G., Pantano P., Totaro P., Calistri V., Colosimo C., Carmellini M., Defazio G., Berardelli A. (2008). Diffusion tensor imaging in patients with primary cervical dystonia and in patients with blepharospasm. Eur. J. Neurol..

[41] Frueh B.R., Felt D.P., Wojno T.H., Musch D.C. (1984). Treatment of blepharospasm with botulinum toxin. A preliminary report. Arch. Ophthalmol..

[42] Tsoy E.A., Buckley E.G., Dutton J.J. (1985). Treatment of blepharospasm with botulinum toxin. Am. J. Ophthalmol..

[43] Karp B.I. (2004). Botulinum toxin treatment of occupational and focal hand dystonia. Mov. Disord..

[44] Cillino S., Raimondi G., Guepratte N., Damiani S., Cillino M., di Pace F., Casuccio A. (2010). Long-term efficacy of botulinum toxin A for treatment of blepharospasm, hemifacial spasm, and spastic entropion: A multicentre study using two drug-dose escalation indexes. Eye.

[45] Costa J., Espirito-Santo C., Borges A., Ferreira J.J., Coelho M., Moore P., Sampaio C. (2005). Botulinum toxin type A therapy for blepharospasm. Cochrane Database Syst. Rev..

[46] Bhidayasiri R., Cardoso F., Truong D.D. (2006). Botulinum toxin in blepharospasm and oromandibular dystonia: Comparing different botulinum toxin preparations. Eur. J. Neurol..

[47] Colosimo C., Tiple D., Berardelli A. (2012). Efficacy and safety of long-term botulinum toxin treatment in craniocervical dystonia: A systematic review. Neurotox. Res..

[48] Lungu C., Karp B.I., Alter K., Zolbrod R., Hallett M. (2011). Long-term follow-up of botulinum toxin therapy for focal hand dystonia: Outcome at 10 years or more. Mov. Disord..

[49] Dressler D. (2010). Botulinum toxin for treatment of dystonia. Eur. J. Neurol..

[50] Lawrence G.W., Ovsepian S.V., Wang J., Aoki K.R., Dolly J.O. (2012). Extravesicular intraneuronal migration of internalized botulinum neurotoxins without detectable inhibition of distal neurotransmission. Biochem. J..

[51] Matak I., Riederer P., Lackovic Z. (2012). Botulinum toxin’s axonal transport from periphery to the spinal cord. Neurochem. Int..

[52] Dressler D., adib Saberi F. (2005). Botulinum toxin: Mechanisms of action. Eur. Neurol..

[53] Trompetto C., Curra A., Buccolieri A., Suppa A., Abbruzzese G., Berardelli A. (2006). Botulinum toxin changes intrafusal feedback in dystonia: A study with the tonic vibration reflex. Mov. Disord..

[54] Abbruzzese G., Berardelli A. (2006). Neurophysiological effects of botulinum toxin type A. Neurotox. Res..

[55] Priori A., Berardelli A., Mercuri B., Manfredi M. (1995). Physiological effects produced by botulinum toxin treatment of upper limb dystonia: Changes in reciprocal inhibition between forearm muscles. Brain.

[56] Naumann M., Reiners K. (1997). Long-latency reflexes of hand muscles in idiopathic focal dystonia and their modification by botulinum toxin. Brain.

[57] Byrnes M.L., Thickbroom G.W., Wilson S.A., Sacco P., Shipman J.M., Stell R., Mastaglia F.L. (1998). The corticomotor representation of upper limb muscles in writer’s cramp and changes following botulinum toxin injection. Brain.

[58] Gilio F., Curra A., Lorenzano C., Modugno N., Manfredi M., Berardelli A. (2000). Effects of botulinum toxin type A on intracortical inhibition in patients with dystonia. Ann. Neurol..

[59] Boroojerdi B., Cohen L.G., Hallett M. (2003). Effects of botulinum toxin on motor system exciatbility in patients with writer’s cramp. Neurology.

[60] Ridding M.C., Sheean G., Rothwell J.C., Inzelberg R., Kujirai T. (1995). Changes in the balance between motor cortical excitation and inhibition in focal, task specific dystonia. J. Neurol. Neurosurg. Psychiatry.

[61] Ceballos-Baumann A.O., Brooks D.J. (1997). Basal ganglia function and dysfunction revealed by PET activation studies. Adv. Neurol..

[62] Kojovic M., Caronni A., Bologna M., Rothwell J.C., Bhatia K.P., Edwards M.J. (2011). Botulinum toxin injections reduce associative plasticity in patients with primary dystonia. Mov. Disord..

[63] Blood A.J., Tuch D.S., Makris N., Makhlouf M.L., Sudarsky L.R., Sharma N. (2006). White matter abnormalities in dystonia normalize after botulinum toxin treatment. Neuroreport.

[64] Valls-Sole J., Tolosa E., Ribera G. (1991). Neurophysiological observations on the effects of botulinum toxin treatment in patients with dystonic blepharospasm. J. Neurol. Neurosurg. Psychiatr.

[65] Conte A., Fabbrini G., Belvisi D., Marsili L., di Stasio F., Berardelli A. (2010). Electrical activation of the orbicularis oculi muscle does not increase the effectiveness of botulinum toxin type A in patients with blepharospasm. Eur. J. Neurol..

[66] Quartarone A., Sant’Angelo A., Battaglia F., Bagnato S., Rizzo V., Morgante F., Rothwell J.C., Siebner H.R., Girlanda P. (2006). Enhanced long-term potentiation-like plasticity of the trigeminal blink reflex circuit in blepharospasm. J. Neurosci..

[67] Zeuner K.E., Knutzen A., Al-Ali A., Hallett M., Deuschl G., Bergmann T.O., Siebner H.R. (2010). Associative stimulation of the supraorbital nerve fails to induce timing-specific plasticity in the human blink reflex. PLoS One.

